# A rare case of *Bacillus subtilis variant natto*-induced persistent bacteremia with liver and splenic abscesses in an immunocompetent patient

**DOI:** 10.1016/j.idcr.2024.e01925

**Published:** 2024-01-14

**Authors:** Tetsuro Amemiya, Kiyofumi Ohkusu, Miku Murayama, Tomokiyo Yamamoto, Naoya Itoh

**Affiliations:** a Department of General Internal Medicine, Aizawa Hospital, 2-5-1 Honjo, Matsumoto-shi, Nagano 390-8510, Japan; b Department of Microbiology, Tokyo Medical University, 6-1-1 Shinjuku, Shinjuku-ku, Tokyo 160-8402, Japan; c Department of Clinical Laboratory, Aizawa Hospital, 2-5-1 Honjo, Matsumoto-shi, Nagano 390-8510, Japan; d Division of Infectious Diseases, Aichi Cancer Center, 1-1 Kanokoden, Chikusa-ku, Nagoya, Aichi 464-8681, Japan

**Keywords:** *Bacillus subtilis*, *Bacillus subtilis variant natto*, Immunocompetent, Persistent bacteremia

## Abstract

*Bacillus subtilis var. natto*, a low-pathogenic bacterium used in the traditional Japanese food "natto" (fermented soybeans), has rarely been reported as a pathogen of infectious diseases in humans. Herein, we report the first case of persistent bacteremia caused by *B. subtilis var. natto* in an immunocompetent patient without any gastrointestinal involvement. A 53-year-old Japanese woman who had been consuming natto every day was admitted to our hospital with complaints of fever and chills. *B. subtilis* was isolated from blood cultures collected during the initial visit. Abdominal contrast-enhanced computed tomography (CT) showed multiple low-absorption areas in the liver and spleen. Treatment commenced with vancomycin; however, *Bacillus sp.* was re-detected in the blood culture on day 4 after treatment initiation. The blood culture on day 8 was negative. Subsequently, the treatment was switched to ampicillin-sulbactam and oral amoxicillin-clavulanic acid, and the patient recovered after 28 days of treatment from the time the blood cultures became negative. Contrast-enhanced CT of the abdomen at the end of treatment showed that the multiple low-absorption areas in the liver and spleen had disappeared. Later, the variant of the bacteria was identified as *B. subtilis var. natto* by DNA analysis. *B. subtilis var. subtilis* and *B. subtilis var. natto* cannot be distinguished using matrix-assisted laser desorption/ionization-time of flight mass spectrometry or 16S rRNA analysis. Biotin auxotrophy of *B. subtilis var. natto* is used to distinguish between the two variants.

## Introduction

**Fig. 1:**
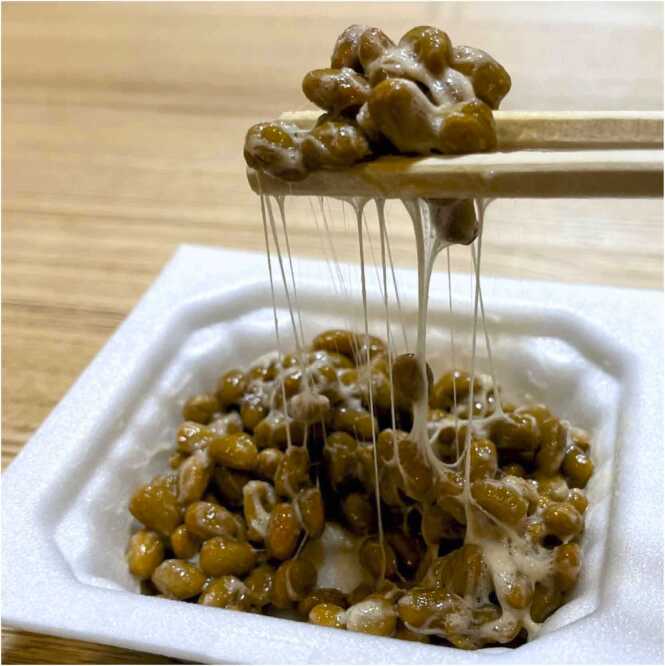
Natto ingested by the patient, Natto is a traditional Japanese food made from steamed and softened soybeans fermented by *Bacillus subtilis var. natto*. It is usually mixed well and becomes stickier the more it is mixed.

*Bacillus subtilis var. natto* is used in the fermentation process of soybeans to make "natto” (Fig. 1), a traditional Japanese fermented food. It is considered healthy when consumed in moderation and has rarely been reported as a causative agent of infectious diseases in humans. Only four cases of bacteremia caused by *B. subtilis var. natto* have been reported previously, all of which were associated with immunodeficiency or gastrointestinal lesions [1], [2], [3], [4]. Here, we report a case of persistent bacteremia caused by *B. subtilis var. natto* in an immunocompetent individual without any gastrointestinal involvement, resulting in liver and spleen abscesses.

## Case

**Fig. 2:**
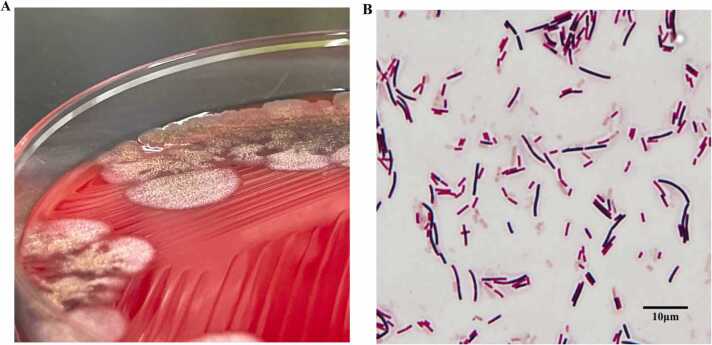
Blood culture findings, **(A)** White-colored colonies of *Bacillus subtilis var. natto* with a rough surface and radial edges are evident on sheep blood agar medium, **(B)** Gram staining of colonies reveals large gram-positive bacilli. Some of the bacteria appear to show partial negative staining (gram variable). Spores are also evident. (Gram staining, ×1000).

A 53-year-old Japanese woman who had been consuming natto every day was admitted to our hospital with complaints of fever and chills for the past two days. She had been prescribed trazodone and magnesium oxide for anxiety and constipation. The patient was diagnosed with anal fissures approximately three months before the onset of symptoms, for which she was treated with topical medication. However, the fissure worsened and bled, and two days later, the patient developed a fever with chills. She was admitted to the emergency room the following day, where two sets of blood cultures were performed, and acetaminophen was prescribed. The blood cultures were positive after 16 h, and Gram staining showed the presence of large gram-positive bacilli. White colonies with rough surfaces and radial edges were evident on sheep blood agar medium (Fig. 2). Based on Gram staining and colony characteristics, the organism was presumed to be *Bacillus sp*. Two days after the initial visit, the patient returned to the emergency room; the fever had resolved by this time. However, blood was collected and retested. The same gram-positive bacilli were detected again, and the patient was hospitalized on the fifth day of illness.

On admission, her vital signs were as follows: body temperature, 36.1 °C (axillary temperature); blood pressure, 128/87 mmHg; pulse rate, 87 beats/min; respiratory rate, 18 breaths/min; and oxygen saturation (SpO_2_), 98% (room air). Her oral mucosa had no erosions or aphthae, no heart murmur was detected, and the abdomen was flat and soft with no tenderness. Bilateral tenderness was not elicited in the bilateral costal areas, and no Osler's nodes or Janeway's regions were observed at the extremities.

**Fig. 3:**
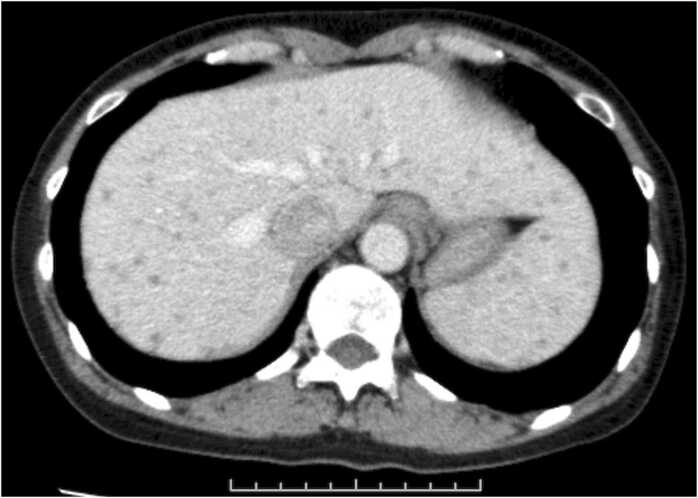
Contrast-enhanced computed tomography (CT) of the abdomen two days before admission, Abdominal contrast-enhanced CT performed two days prior to admission shows multiple small, low-absorption areas in the liver and spleen.

Laboratory investigation showed mild inflammation, with a white blood cell count of 3610/µL, and a C-reactive protein level of 0.86 mg/dL. The qualitative test result for the human immunodeficiency virus antigen-antibody was negative. No decrease in immunoglobulins was observed (IgG, 1213 mg/dL; IgA, 355 mg/dL; IgM, 56 mg/dL). Contrast-enhanced computed tomography (CT) of the abdomen showed multiple small non-contrast areas in the liver and spleen (Fig. 3). Gram-positive bacilli isolated from the blood culture at the initial visit were later identified as *B. subtilis* by matrix-assisted laser desorption/ionization-time of flight mass spectrometry (MALDI-TOF MS) (Bruker MALDI Biotyper®, library Version 4.1.100) (score value: 2.10).

The susceptibility results based on CLSI M45-Ed3 showed vancomycin and ampicillin sensitivity (Table 1).

**Table 1:** Antimicrobial susceptibility of *Bacillus subtilis* isolated from blood cultures (according to CLSI M45-Ed3).

	MIC (μg/mL)	Susceptibility
PCG	<0.03	S
ABPC	<0.12	S
CLDM	<0.5	S
EM	<0.25	S
GM	<1	S
IPM	<1	S
LVFX	<0.5	S
RFP	<1	S
ST	<1	S
VCM	<0.5	S

Abbreviations: S, susceptible; MIC, minimum inhibitory concentration. ABPC, ampicillin; CLDM, clindamycin; EM, erythromycin; GM, gentamycin; IPM, imipenem; LVFX, levofloxacin; PCG, penicillin G; RFP, rifampicin; ST, sulfamethoxazole-trimethoprim; VCM, vancomycin

The isolates were examined to determine the variant. DNA analysis showed that both *BioF* and *BioW* regions of the isolates were 100% homologous to the *B. subtilis var. natto* standard strain. Compared to the *B. subtilis var. subtilis* standard strain, the *BioF* region of the isolate had approximately 50 fewer bases, and the *BioW* region had a base mutation that resulted in a termination codon for amino acid synthesis. These results indicated that the isolate was *B. subtilis var. natto*.

Vancomycin 2 g/day treatment was initiated after admission. After hospital admission, the patient did not develop a fever. Transthoracic echocardiography performed on the day of admission showed no vegetations. On the fourth day of hospitalization, blood cultures were retested, and the same gram-positive bacilli were detected again. Transesophageal echocardiography performed on day 6 of hospitalization also showed no vegetations. On day 8 of hospitalization, the blood cultures were retested and confirmed to be negative. A lower gastrointestinal endoscopy was performed on the same day, which revealed no mucosal lesions other than an anal fissure. Upper gastrointestinal endoscopy performed on day 9 of admission revealed no mucosal lesions other than gastric fundic gland polyps. On day 19 of hospitalization, the patient was switched to ampicillin-sulbactam 12 g/day. On day 23 of hospitalization, oral amoxicillin-clavulanic acid 1500 mg/day was commenced. The patient was discharged on day 24 of hospitalization. Amoxicillin-clavulanic acid was continued for 13 days and administered for a total of 36 days (28 days from the time of the negative blood culture) before treatment was terminated. Contrast-enhanced CT of the abdomen after termination of treatment revealed that the contrast-impaired areas of the liver and spleen had disappeared. The patient was asymptomatic 15 days after treatment ended and was given a final medical consultation, where it was confirmed that the infection had not recurred.

## Discussion

Here, we report a case of persistent bacteremia with hepatic and splenic abscesses caused by *B. subtilis var. natto* in an immunocompetent patient. Very few reports exist on *B. subtilis var. natto* causing infections in humans. We believe this case is noteworthy as it shows that a less virulent bacterium, such as those used in foods, can result in bacteremia in patients without immunodeficiency and gastrointestinal involvement. Moreover, no previous studies have reported persistent bacteremia or liver or spleen abscesses associated with *B. subtilis var. natto*.

*B. subtilis var. subtilis* and *B. subtilis var. natto* cannot be distinguished using automated measuring devices or MALDI-TOF MS. 16 S rRNA analysis also cannot distinguish between the two. Unlike *B. subtilis var. subtilis*, *B. subtilis var. natto* is characterized by a lack of biotin synthesis ability, and the two can be distinguished by analyzing the gene responsible for biotin synthesis ability [5]. The genes involved in biotin synthesis in *B. subtilis* are *BioF* and *BioW.* However, *B. subtilis var. natto* cannot synthesize biotin due to mutations in the *BioF* and *BioW* regions [6]. Herein, the infectious pathogen was proven to be *B. subtilis var. natto* by analyzing the *BioF* and *BioW* regions. Differences in colony characteristics were also observed. *B. subtilis var. subtilis* tends to form relatively unstructured colonies, whereas *B. subtilis var. natto* is characterized by a radial structure with chrysanthemum-like edges, especially observed in fresh isolates [7].

Only four case reports of bacteremia caused by *B. subtilis var. natto* are present in the literature. Aoyagi et al. reported a case of *B. subtilis var. natto* bacteremia that developed in an individual with immunodeficiency while receiving glucocorticoids and tocilizumab for coronavirus disease 2019 [1]. Since *B. subtilis var. natto* is a less toxic bacterium, it is thought that reduced immunity puts patients at risk of developing the disease. Tanaka et al. [2] and Hashimoto et al. [3] reported cases of *B. subtilis var. natto* bacteremia in patients with gastrointestinal perforation, whereas Tokano et al. reported a case of *B. subtilis var. natto* bacteremia with bacterial meningitis in a patient with erosive esophagitis with hematemesis [4]. Since the bacteria enters the gastrointestinal tract via oral route, it is likely that gastrointestinal lesions were the source of bacteremia. *B. subtilis* bacteremia in patients in Japan has been associated with a history of recent natto inoculation [8], suggesting that some of the *B. subtilis* bacteremia cases reported in Japan may have been caused by natto bacteremia. Although it was not mentioned whether it was *B. subtilis var. natto*, several cases of *B. subtilis* bacteremia in Japan had the gastrointestinal tract as the entry route [9]. In our case, no obvious gastrointestinal lesions were noted, but the disease onset occurred two days after bleeding from the anal fissure; thus, we speculate that the bacteria entered thought the anal fissure.

Unlike *B. cereus*, *B. subtilis* detected in Japan shows good penicillin susceptibility [8], [9], [10]. However, *B. subtilis* detected in Iraq showed relatively poor susceptibility to penicillin [11]. Tanaka et al. commenced treatment with piperacillin-tazobactam, used teicoplanin during the course of treatment, and then switched to ampicillin-sulbactam. The patient recovered after 39 days of antimicrobial therapy [2]. Although not limited to *B. subtilis var. natto*, the National Center for Global Health and Medicine reported that all four cases of *B. subtilis* bacteremia treated with a β-lactam/β-lactamase inhibitor combination were cured completely [9]. Although sensitivity to ampicillin was seen in the current case, ampicillin-sulbactam was selected, and the patient was cured. Previous case reports have reported using a β-lactam/β-lactamase inhibitor combination instead of penicillin alone. *B. subtilis* bacteremia occurring in Japan, including that due to *B. subtilis var. natto,* can be treated with penicillin. Herein, after switching from vancomycin to penicillin, liver and spleen abscesses disappeared.

In conclusion, bacteremia caused by *B. subtilis var. natto* can occur even in immunocompetent patients due to the entry of the pathogen via small gastrointestinal lesions. Several *B. subtilis* bacteremia cases reported in the past in Japan may have been due to *B. subtilis var. natto*. Bacteremia caused by *B. subtilis var. natto* can be treated with penicillin.

## Consent for publication

Written informed consent was obtained from the patient for the publication of this case report and accompanying images. A copy of the written consent form is available for review by the editor of this journal.

## Funding

This study did not receive any specific grants from funding agencies in the public, commercial, or not-for-profit sectors.

## Authors’ contributions

TA and NI drafted the manuscript. TA and TY were involved in patient care. TA planned the study and reviewed the literature. KO and MM conducted laboratory analyses. NI was involved in supervision. All authors interpreted the data, drafted and critically revised the manuscript, and approved its final version.

## Declaration of Competing Interest

The authors have no conflicts of interest to declare.

## Data Availability

The data used and/or analyzed during this study are available from the corresponding author upon reasonable request.
